# AI-augmented communication improves HIV PrEP initiation and persistence in populations disproportionately impacted by HIV

**DOI:** 10.1038/s41746-026-02519-3

**Published:** 2026-03-07

**Authors:** Aditya Narayan, Michael Blasingame, Sam Warmuth, Gabriella Palmeri, India Halm, Ramin Bastani, Whitney Engeran-Cordova, Harold J. Phillips, Leandro Mena, Nirav R. Shah

**Affiliations:** 1 Clinical Excellence Research Center, Stanford University, Palo Alto, CA USA; 2 Healthvana Inc, Los Angeles, CA USA; 3 AIDS Healthcare Foundation, Los Angeles, CA USA; 4 National Minority AIDS Council, Washington, DC USA; 5 Emory University, Atlanta, GA USA

**Keywords:** Diseases, Health care, Medical research

## Abstract

This retrospective cohort study evaluated an AI-powered chatbot used for PrEP support across AIDS Healthcare Foundation clinics in the United States. Among 155,217 eligible adults, individuals who engaged with the chatbot had higher rates of PrEP initiation, follow-up attendance, and appointment adherence than non-users. Engagement was greatest among younger and racial or ethnic minority patients. Findings suggest that AI-supported communication may enhance aspects of PrEP care delivery.

HIV remains a pressing global and domestic public‑health challenge: in 2023, an estimated 39.9 million people worldwide—and roughly 1.2 million in the United States—were living with the diagnosis, while 1.3 million new infections occurred globally that year^1–3^. In the US, incident cases continue to cluster disproportionately among Black, Latino, and LGBTQ+ communities, underscoring persistent disparities in prevention and care. Each infection carries considerable human and economic costs: untreated HIV progresses to AIDS and premature mortality, and even with modern antiretroviral therapy, an individual faces lifelong clinical follow‑up, with recent real‑world claims data projecting discounted cumulative direct medical costs of about $984,000 (2017 USD) and undiscounted costs exceeding $1.8 million per person aged 25–69 years^4^. As these figures climb, the imperative to prevent new infections intensifies—particularly given the widening availability of technological solutions that can scale prevention. Recent advances in telehealth, mobile applications, and AI-driven conversational systems integrated with electronic health records enable real-time counseling, coordinated care, and individualized prevention support across large populations. When applied thoughtfully, digital communication tools such as large language models (LLMs) may enhance PrEP engagement and continuity of care.

In 2019, the US Department of Health and Human Services (HHS) announced its plan, “Ending the HIV Epidemic in the US by 2030”, to drastically reduce new HIV diagnoses, with the goal of cutting new HIV cases by 75% by 2025 and 90% by 2030^5^. Key strategies include: diagnosing all individuals with HIV as early as possible after infection and preventing new HIV transmissions by using proven interventions, including pre-exposure prophylaxis (PrEP)^5^.

PrEP is a highly effective biomedical intervention for preventing HIV infection^6^. PrEP refers to the use of antiretroviral medications by HIV-negative individuals to avert HIV acquisition before potential exposure. Two oral PrEP regimens are currently approved by the FDA: a combination of tenofovir disoproxil fumarate and emtricitabine (TDF/FTC), and a combination of tenofovir alafenamide and emtricitabine (TAF/FTC) (not approved for those assigned female at birth)^7^. When used as prescribed, daily oral PrEP has been shown to reduce HIV acquisition by up to ~99% from sexual exposure, and ≥74% from injection drug use; additionally, event-driven (on-demand) oral PrEP has demonstrated high effectiveness among some men who have sex with men^7^. Widespread implementation of PrEP—alongside other prevention strategies such as consistent condom use, post-exposure prophylaxis (PEP), sterile needle and syringe programs, and treatment-as-prevention—is recognized by public health authorities as a cornerstone of efforts to end the HIV epidemic and reduce long-term healthcare burdens.

Despite PrEP’s proven efficacy, its real-world impact has been limited by suboptimal uptake and persistence. In 2020, an estimated 300,000 people—roughly 25% of those with a PrEP indication in the US—were prescribed PrEP^8^. Uptake has been especially low in communities disproportionately affected by HIV; for example, among Black individuals who could benefit, PrEP coverage was roughly 9% in 2020. Moreover, many PrEP users face persistence challenges with the medication, as a consequence of patient-level, system-level, and economic barriers, thereby^9–13^.

Patient-level factors—including stigma arising from the misconception that PrEP use implies promiscuity or an HIV diagnosis, medical mistrust rooted in historical and ongoing discrimination, low health literacy, and competing life priorities such as housing instability and mental health comorbidities—have been shown to discourage both initiation and sustained use of PrEP^14,15^. Structural barriers further impede access: uneven geographic distribution of PrEP providers, inflexible clinic hours, and high no-show rates driven by transportation and scheduling conflicts; provider inertia due to limited time, training, or comfort in discussing HIV prevention; and bureaucratic requirements for insurance authorization and quarterly laboratory monitoring all contribute to gaps in PrEP delivery^13^. Economic obstacles compound these challenges, as out-of-pocket medication costs, co-payments for clinic visits and laboratory services, and ancillary expenses (transportation, time off work) can be prohibitive for many patients, despite the availability of generics and assistance programs. Addressing this triad of patient-, system-, and economic-level barriers is critical to improving PrEP uptake and persistence.

The aforementioned barriers have led to interest in innovative interventions to improve PrEP engagement, including digital health tools. Short message service (SMS) reminders represent one of the earliest digital strategies to promote treatment persistence. Studies in HIV treatment and prevention have shown that daily or weekly SMS reminders can modestly improve adherence for some patients, likely by providing a prompt or cue to take medication^16^. However, the impact of basic SMS programs on the long-term persistence of medication use has been mixed. For example, A youth‑tailored, bidirectional SMS system (PrEPmate) subsequently raised both visit retention (86% vs 71%) and pharmacologic persistence (72% vs 57%) in a randomized study of young MSM in Chicago’s safety‑net clinics^17^. Mobile apps have also been developed to support PrEP users. These apps often include features like daily pill-taking checklists, refill reminders, educational modules about HIV and PrEP, risk assessment tools, and peer support forums^18–20^. The expansion of telemedicine in recent years—accelerated by the COVID-19 pandemic—has opened new avenues for PrEP delivery. “TelePrEP” programs enable patients to engage in PrEP care (initial consultations, prescription refills, and follow-up counseling) remotely via phone or video, without needing frequent in-person clinic visits. TelePrEP has been especially useful for reaching rural populations and others with limited physical access to PrEP providers^21^. Several telehealth-based PrEP services in the US have demonstrated high patient satisfaction and uptake^22^.

More recently, advanced LLM platforms have made it feasible to build chatbots that converse fluidly with patients, interpret free‑text questions, and generate context‑aware answers. Pilot deployments span mental‑health coaching, chronic‑disease medication reminders, and basic patient‑service triage, but rigorous evidence of downstream clinical or population‑level benefit remains sparse. A subsequent 2020 meta‑review that included 31 studies reached a similar conclusion, noting generally positive usability signals but “insufficient quality of evidence” to support impacts on health metrics, cost‑effectiveness, or safety^23^. A 2024 rapid review echoed these concerns, highlighting pervasive limitations in study design, short follow‑up, and the absence of standardized outcome reporting, and called for more robust trials before chatbots can be integrated confidently into routine care^24^. Against this backdrop, we examined an AI‑driven PrEP chatbot embedded in AIDS Healthcare Foundation (AHF) Wellness clinics across 15 states. Unlike most prior studies limited by small sample sizes, brief follow-up, or simulated interactions, this large-scale real-world implementation assessed longitudinal outcomes across a large sample set.

## Methods

A retrospective cohort study was conducted across AHF Wellness clinics in the United States following the launch of a conversational AI-based messenger developed by the private company, Healthvana, in January 2024.

### Study population

During the study period (January 1, 2024—April 29, 2025), all Healthvana patient accounts linked to AHF clinics were evaluated for eligibility (approximately 160,000 total records). Individuals younger than 18 years (*n* = 2274) or known to be living with HIV (*n* = 1356) were excluded, as Healthvana does not process HIV-positive results and such clients are referred to specialized care. After exclusions, 155,217 HIV-negative adults with at least one clinic visit during the study period were included in the analytic cohort.

Every individual with an active account on the Healthvana patient-messaging platform automatically receives access to the AI-enabled chatbot. Users were informed of this access via direct electronic notification within the Healthvana system, which is routinely used by AHF clinics for appointment reminders and test result communication. The intervention was deployed uniformly across all participating clinics, with no site-specific recruitment, posters, or staff-mediated enrollment. All eligible clients automatically received access and were informed via standardized notifications: (1) a text message sent at clinic registration, and (2) a single email announcement. AHF represents a group of free-standing, community-based sexual health centers operated by a private nonprofit organization. AHF Wellness Clinics provide low- or no-cost services, including HIV testing, PrEP counseling, and preventive care, to both insured and uninsured individuals across 15 states (Arizona, California, Florida, Georgia, Illinois, Louisiana, Maryland, Mississippi, Nevada, New York, Ohio, Pennsylvania, South Carolina, Texas, Washington, and additionally Washington, D.C.).

Eligibility criteria were defined retrospectively using the CDC’s Clinical Guidance for PrEP (updated February 2025), which reflects current US recommendations and is consistent with prior guidance from 2021. Under this framework, PrEP is recommended for adults and adolescents aged ≥ 16 years without HIV who may be exposed through sex or injection-drug use, as well as for any individual requesting PrEP regardless of disclosed risk factors^25^. Eligible individuals were HIV-negative, had a documented negative HIV test and baseline safety laboratories (e.g., renal function) before PrEP initiation, and attended at least one AHF wellness clinic visit between 1 January 2024 and 29 April 2025. Two cohorts were defined: Individuals who engaged in at least one message exchange (read or sent) were classified as “AI users,” whereas those with portal access but no recorded interactions were classified as “non-users. Individuals with documented PrEP prescriptions before 1 January 2024 were excluded from analyses of PrEP initiation and retention to focus on new PrEP users.

### Human ethics declaration

This study was reviewed by the Advarra Institutional Review Board, which determined that the project does not meet the definition of human subjects research under 45 CFR 46 and is therefore exempt from IRB oversight (Protocol ID: Pro00084144; determination dated January 3, 2025). The study involved retrospective analysis of de-identified data and did not involve contact with participants. Informed consent was not required.

### Consent to participate

Individual informed consent was not required, as the study involved retrospective analysis of de-identified quality improvement data. The project received a non-human subjects research determination from the Advarra Institutional Review Board.

### Intervention

The study period began January 1, 2024, coinciding with the deployment of the conversational AI across all AHF clinics. AHF clients who did not have a visit after January 1, 2024, were not included in the study. Healthvana deploys its HIPAA‑compliant Conversational AI by first executing a Business Associate Agreement with OpenAI that mandates zero retention of protected health information, then evaluating its performance against the White House “FAVES” (fair, appropriate, valid, effective, safe) principles. Patients are notified that responses are AI‑generated and subject to optional human review. The system draws from the electronic health record to answer logistical and prevention‑focused queries, presents content at a sixth‑grade reading level in roughly 50 languages, and offers two selectable personas (“Standard” and “Glitter Byte,” a humorous and upbeat “drag-queen” persona which uses affirming language drawn from the LGBTQ+ community). Of note, the AI chatbot was not designed to provide individualized clinical guidance or diagnostic recommendations. Instead, it delivered standardized, evidence-informed education and behavioral reminders related to PrEP adherence, appointment scheduling, and general sexual health.

Because the underlying model’s training data stop at September 2023, and it has no internet access, domain experts periodically inject updated clinical information (e.g., Doxy‑PEP, long‑acting injectable PrEP), organization‑specific policies, and de‑stigmatizing language rules. Of note, however, during the study period, the AI chatbot supported only oral PrEP management. Quality is safeguarded through two layers: an automated replay framework that stress‑tests thousands of prompt–response permutations after each code change, and near-real‑time human scoring of every outbound AI message against a five‑point rubric for accuracy, comprehensiveness, and contextual sensitivity, with more than 100 AHF providers and trained staff contributing feedback during development. AI-generated responses receiving a score of 2 or lower were subject to further human review (Supplementary Table 1).

Human reviewers consisted of trained staff who were certified in PrEP navigation and patient support. Several reviewers were bilingual and conducted evaluations in both English and Spanish to ensure equitable language coverage. All raters completed a structured onboarding program that included review of CDC PrEP guidelines, a medical accuracy training led by an infectious disease specialist (L.M.), and certification on Healthvana’s internal scoring rubric, which was developed by Healthvana based on Stanford’s HELM framework. During calibration, reviewers compared sample AI responses with gold-standard clinician answers until scoring agreement exceeded 90%. Each reviewer was provided a concise reference guide summarizing current PrEP and sexual health protocols to support consistency in factual accuracy checks. When an AI message was judged inaccurate or incomplete, the instance was logged within Healthvana’s internal review platform and routed for secondary evaluation by secondary raters within 24 h. The secondary reviewer determined whether a clarification to the patient was warranted and, if needed, updated the knowledge base or prompt library. This process ensured that every flagged message resulted in both a real-time correction for the patient and a systemic update to prevent future recurrence.

### Outcomes studied

Primary outcomes were (1) PrEP initiation—defined as a new PrEP prescription documented after Jan 1, 2024; (2) PrEP retention—defined as having at least one follow-up PrEP care visit after initiation that resulted in a prescription refill (beyond the initial prescription); and (3) PrEP appointment adherence—defined as attending a scheduled PrEP clinic appointment (versus no-show) after receiving an AI-generated reminder. The appointment-reminder feature of the AI chatbot was introduced on 1 May 2024 and evaluated for all subsequent PrEP-related visits during the study period. Secondary outcomes included patient-AI engagement metrics (message volumes, session lengths, persona preferences), patient demographic patterns, and staff-reported workload changes. Engagement metrics were calculated as the total number of messages exchanged during each user’s period of AI access (rather than within a fixed time interval). Because the Conversational AI sends informational messages regardless of user interaction (e.g., PrEP cycle starts; appointment reminders, etc.), we categorized users into two groups: those who viewed AI messages and those who did not.

### Data sources

De-identified data were extracted from the AHF electronic health record and the Healthvana platform, including prescription dates, visit dates, appointment statuses, and AI interaction logs. Demographic variables (age, sex assigned at birth, race/ethnicity, geographic region) were obtained from patient registration records. Clinic staff feedback on workflow impact was collected via an internal survey six months after conversational AI implementation. To evaluate perceived workflow impact, clinic staff completed an internal online survey six months after AI implementation. The survey included both structured and open-ended questions covering clinic location, perceived changes in workload (“How has the Conversational AI changed your workload?”), patient impact (“What effect has the Conversational AI had on patients?”), and perceptions of patient experience (“Do you think the AI is changing how patients feel about AHF?”). Staff were also invited to provide free-text comments describing observed effects and suggestions for improvement. Responses were analyzed descriptively to identify common themes regarding workload efficiency and patient engagement.

### Statistical analysis

Because only de-identified demographic fields were available (age, gender identity, race/ethnicity, and region), analyses were limited to unadjusted comparisons between AI users and non-users. Because these fields are required for registration and care documentation, there was no missing data for the included variables. All eligible clients during the study period were included to minimize selection bias. Risk ratios and chi-square tests were used to estimate crude associations; multivariable modeling was not possible due to the absence of individual socioeconomic, behavioral, or clinic-level covariates. Patient demographics were compared across groups by calculating standardized mean differences (SMDs). Comparisons between AI-interaction and non-interaction cohorts were conducted using two-sample tests for proportions (χ² tests), with risk ratios estimating the relative likelihood of each outcome. Two-sided *p* < 0.05 was considered statistically significant. Engagement metrics were summarized descriptively. For individuals who completed at least one PrEP follow-up visit, we calculated the interval in days between the index visit and the first follow-up. Group differences (AI-interacted vs non-interacted) were evaluated using independent-samples *t*-tests with Welch’s correction. Distributional assumptions were assessed by histogram and *Q*–*Q* plot inspection and the Shapiro–Wilk test. To complement mean comparisons under modest deviations from normality, we also report median and interquartile range (IQR). SMDs were expressed as Cohen’s *d*. Two-sided α = 0.05.

### Study outcomes

#### Participants

A total of 155,217 HIV-negative adults met the inclusion criteria of no prior PrEP before 2024 with at least one clinic visit during the study period (Table 1). Notably, the majority were from Ending the HIV Epidemic jurisdictions (geographic regions in which a majority of HIV diagnoses occur. Communities of color were heavily represented among AI users: 80.7% identified as Black/African American, Hispanic/Latino, Asian, or multi-racial. In comparing demographic characteristics across groups, SMDs were below 0.10 (maximum = 0.06), indicating that demographic characteristics were highly comparable between AI users and non-users. The highest SMD was for the age group at 0.06, reflecting a small difference between cohorts. Gender identity showed an SMD of 0.04, while race and ethnicity had an SMD of 0.03. Collectively, these values indicate that the two groups were demographically similar, and any differences are not practically meaningful.

**Table 1:** Demographic distribution of race/ethnicity, gender identity, and age group among patients eligible for PrEP at AIHF clinics from January 1, 2024, to April 29, 2025

	Did not interact with AI *n* = … (…%)	interacted with AI *n* = … (…%)
Race/ethnicity		
American native/Pacific Islander	552 (0.5%)	147 (0.4%)
Asian	4511 (3.8%)	1367 (3.8%)
Black/African American	51,027 (42.9%)	15,639 (43.0%)
Declined	5859 (4.9%)	1274 (3.5%)
Hispanic or Latino	29,144 (24.5%)	9805 (26.9%)
Mixed race/other	6877 (5.8%)	1966 (5.4%)
White/Caucasian	20,853 (17.5%)	6196 (17%)
Gender		
Declined	873 (0.7%)	81 (0.2%)
Female	44,598 (37.5%)	13,946 (38.3%)
Male	70,356 (59.2%)	21,465 (59%)
Non-binary	97 (0.1%)	28 (0.1%)
Other	1753 (1.5%)	488 (1.3%)
Transgender	1146 (1%)	386 (1.1%)
Age group		
18–29	57,118 (48.1%)	17,444 (47.9%)
30–39	41,336 (34.8%)	12,011 (33.0%)
40–49	12,972 (10.9%)	4272 (11.7%)
50–59	4886 (4.1%)	1763 (4.8%)
60+	2511 (2.1%)	904 (2.5%)
US region of residence		
South	78,621 (66.2%)	24,137 (66.3%)
West	27,864 (23.3%)	8349 (22.9%)
Midwest	8029 (6.8%)	2577 (7.1%)
Northeast	4309 (3.6%)	1331 (3.6%)
Overall group total	118,823	36,394
Grand total	155,217	

#### PrEP initiation

**Fig. 1:**
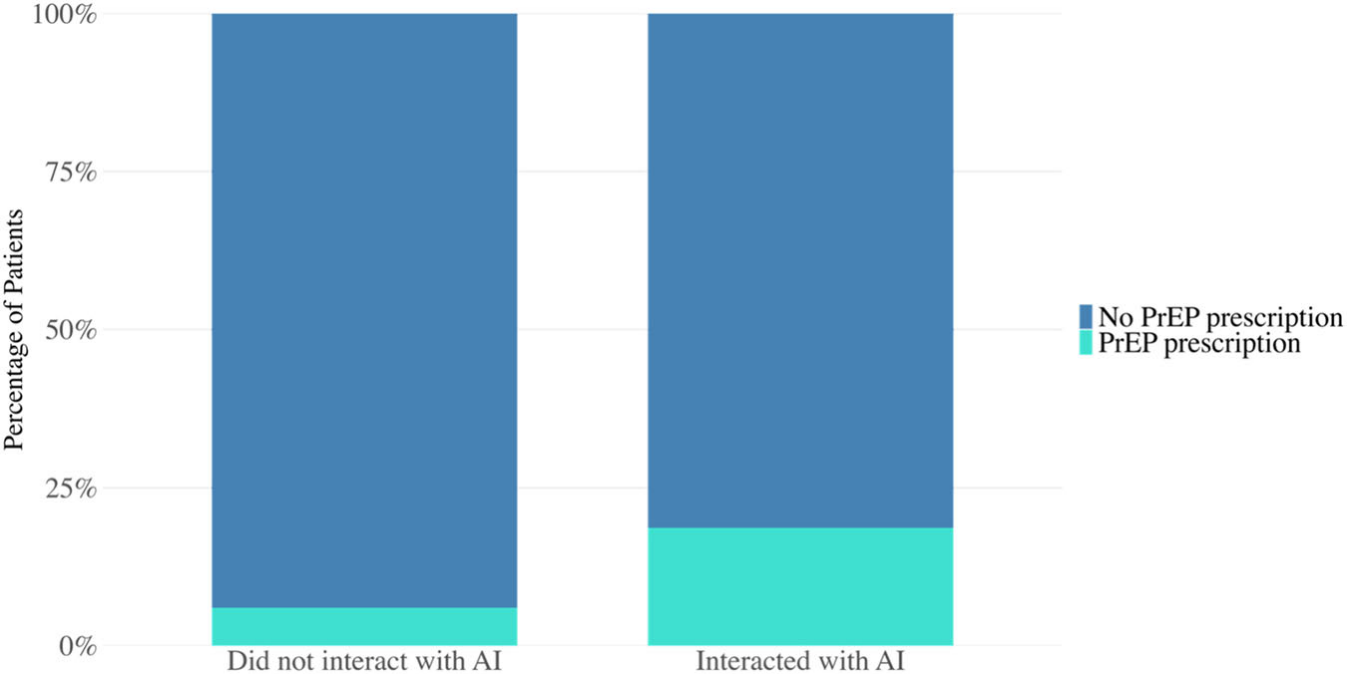
PrEP initiation outcomes with vs without the AI chatbot intervention. Stacked bar charts depict the proportion of those who obtained a PrEP prescription (teal segment) versus those who did not (blue segment) among patients interacting with the AI (right) and those with no AI interaction (left). Individuals who engaged with the AI had a PrEP initiation rate of 19.3%, approximately triple the rate of 6.1% in those who did not use the AI. This difference was highly significant (χ² = 5765.88, *p* < 0.0001).

Among individuals with no prior PrEP use (*n* = 155,217), 36,394 (23.4%) interacted with the AI chatbot and 118,823 (76.6%) did not (Fig. 1). New PrEP prescriptions were documented in 7012 of 36,394 AI users (19.3%) versus 7268 of 118,823 non-users (6.1%). This corresponds to a risk ratio of 3.15 (95% CI: 3.00–3.27; *p* < 0.0001).

#### PrEP persistence (follow-up visits)

**Fig. 2:**
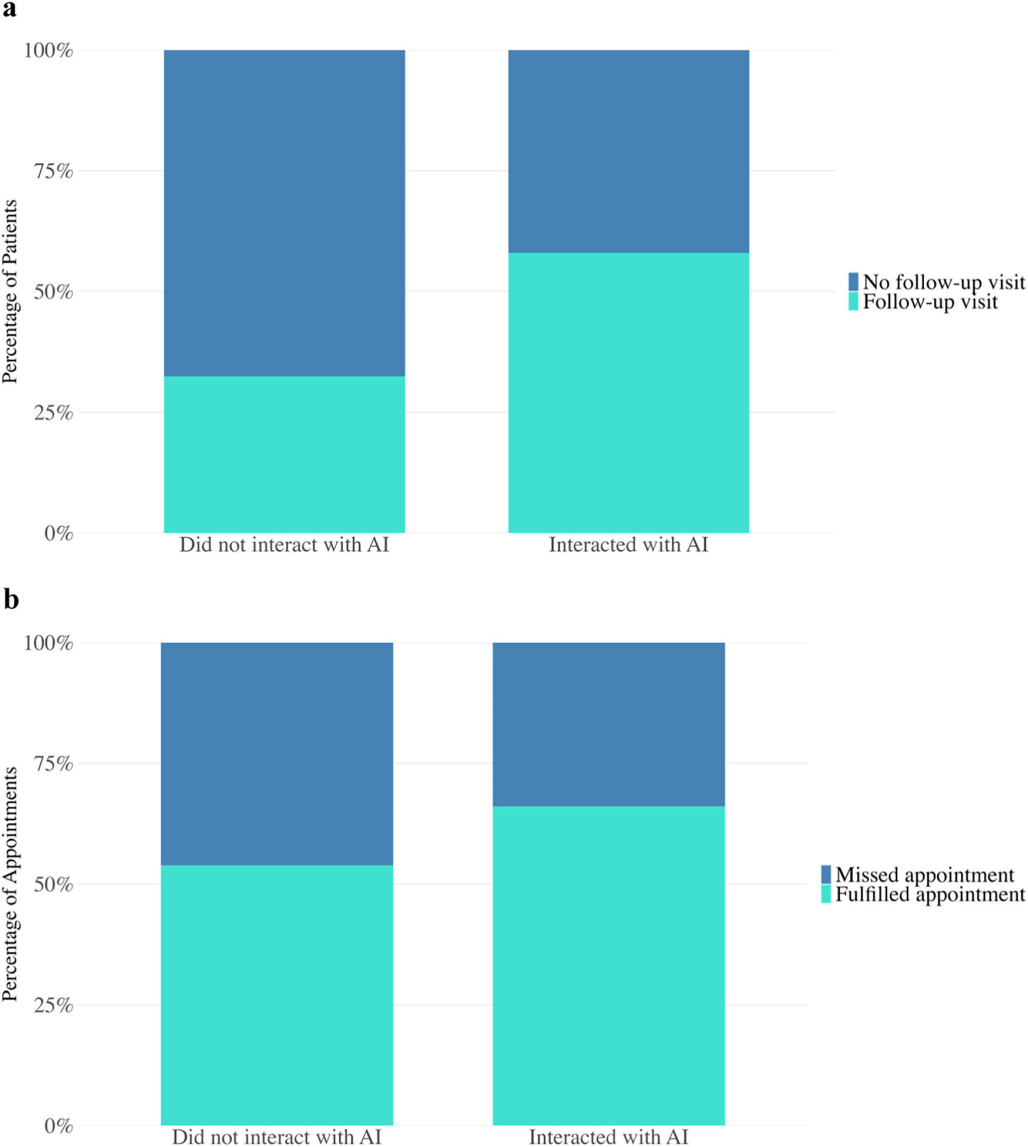
AI engagement and reminder features in relation to PrEP follow-up and appointment adherence. **a** PrEP Persistence (follow‑up visit) rates among new PrEP users, comparing those who interacted with the AI tool versus those who did not. “Return Visit” (teal) denotes individuals who returned for at least one PrEP follow‑up visit resulting in a prescription refill after initiation. “No Return Visit” (blue) denotes individuals who were lost to follow‑up. PrEP retention was 57% with AI versus 32% without AI (*p* < 0.0001). **b** PrEP appointment attendance with vs without AI-generated reminders. Blue represents the proportion of scheduled PrEP appointments that were attended (“fulfilled”), and teal represents appointments where the patient did not attend. With AI reminders, 66% of appointments were attended, compared to 54% attendance without AI. This corresponds to a no-show rate of 34% with AI vs 46% without AI (χ² = 219.8, *p* < 0.0001).

Engagement with the conversational AI was associated with substantially improved short-term retention in PrEP care (Fig. 2). Among 13,831 individuals who initiated PrEP in 2024, 3841 of 6623 AI users (57.0%) returned for at least one documented follow-up visit or prescription refill, compared with 2340 of 7208 non-users (32.4%). This corresponds to a 1.8-fold higher rate of follow-up among AI users (RR = 1.78; χ² = 913.9; *p* < 0.0001).

#### Appointment attendance

We analyzed 15,515 appointments that occurred after this feature launched (May 1, 2024). Appointment attendance was higher among individuals who interacted with the AI tool (66.1%) compared with those who did not (53.9%; *p* < 0.0001) (Fig. 2b). Clinic staff reported that individuals often replied interactively to the AI reminder (e.g., confirming or requesting to reschedule), which helped reduce missed visits.

Among return-visit clients, AI users (*n* = 3841) returned sooner than non-users (*n* = 2340): 65.1 days (SD 35.5), median 64.5 (IQR: 47.2) vs 79.0 days (SD 61.4), median 80.1 (IQR: 81.5). Distributions were approximately normal by Shapiro–Wilk (AI: *W* = 0.9996, *p* = 0.636; non-AI: *W* = 0.9994, *p* = 0.612) with visual confirmation. Welch’s *t*-test indicated a significant mean difference of 14.0 days (95% CI: 11.22–16.68; *t* = 10.02, d*f* = 3307.4, *p* < 0.001), with a small-to-moderate effect size (Cohen’s *d* = 0.30, 95% CI: 0.245–0.348). For individuals with at least two visits, AI users had a median follow-up interval of 208 days (IQR: 81–347), compared with 146 days among non-users (IQR: 29–276), indicating longer follow-up duration among AI-engaged patients. Additionally, among individuals with at least one follow-up visit during the study period, AI users had a median of 3 visits (IQR: 2–5), while non-users had a median of 2 visits (IQR: 2–3), indicating a higher number of visits for AI-engaged clients. With respect to prescriptions, for individuals with at least one prescription, AI users had a median of 5 monthly prescriptions (IQR: 2–10), compared with a median of 2 monthly prescriptions among non-users (IQR: 1–5), indicating higher prescription frequency among AI-engaged individuals.

#### Patient–AI engagement metrics

Over the study period, users exchanged more than 50,000 messages related to PrEP and broad sexual health. The median number of messages per user was 5 (IQR: 2–15), and approximately 25% of users exchanged more than 50 messages. A subset of participants (*n* = 30) engaged in over 100 message exchanges; the most extensive single session comprised 120 messages over 2.5 h. Of individuals who sent at least one message to their provider, AI users had a median of 3 messages (IQR: 1–7), compared with a median of 2 messages among non-users (IQR: 1–3).

Persona selection data indicated that 80% of users opted for the enhanced (“Glitter Byte”) persona rather than the standard clinical tone. Chat transcripts demonstrated the use of 18 different languages, with Spanish as the second most common after English. Sensitive topics—such as condomless sex, new sexually transmitted infection symptoms, anxiety regarding HIV testing, experiences of sexual assault, and questions about sexual orientation—were discussed via the AI. In 15 instances, the chatbot detected language suggestive of severe distress (for example, expressions of suicidal ideation or trauma) and automatically escalated these cases to a human care team member in accordance with established protocols.

#### Staff workload and operational impact

An internal survey conducted six months after AI implementation (*n* = 32 clinic staff, including providers, nurses, and counselors) found that 56% of respondents reported saving time on routine patient communications and follow-up tasks. Analysis of message traffic corroborated these perceptions: by the end of the study period, the AI chatbot addressed approximately 50% of all inbound patient messages to the clinic system, serving as a first-line navigator for appointment scheduling, medication refill inquiries, and common side-effect questions. Notably, 29.8 of messages were generated outside of clinic hours. Clinic managers noted improvements in the timeliness of follow-up for abnormal laboratory results, attributing these gains to the AI’s automated reminders for lab visits and prescription renewals.

### Interpretation of findings

In this large retrospective cohort study, an AI-powered chatbot intervention was associated with marked improvements in key PrEP outcomes among a diverse population. To our knowledge, this is the first report demonstrating that a conversational AI agent supported by human review can significantly improve real-world HIV prevention outcomes at scale. These findings have important implications for public health practice and suggest that leveraging AI for personalized patient engagement has the potential to narrow gaps in the HIV PrEP continuum^26,27^.

The demographic composition of the cohort—predominantly male, and with 80.7% from racial/ethnic minority groups—reflects the populations most affected by HIV in the United States^28,29^. The high uptake of the AI tool among communities of color suggests that customized, culturally sensitive digital interventions may achieve broader adoption in those most affected by HIV, addressing a critical equity gap in PrEP implementation. It may also be the case that the AI tool was perceived as less stigmatizing by communities experiencing a greater degree of anticipated and actual stigma^30^.

Those who engaged with the AI chatbot initiated PrEP at a significantly higher rate than those who did not. For context, the US PrEPTECH telehealth trial—which randomized young sexual and gender‑minority adults to a web‑based PrEP platform versus online resource links—reported greater self‑reported PrEP initiation at 90 days in the intervention arm (odds ratio 6.63; 95% CI: 2.54–17.35), although absolute initiation proportions were not presented^31^. At the other end of the resource spectrum, the client‑centered care‑coordination model used in HPTN 073 achieved 79% PrEP initiation among 226 Black men who have sex with men in three US cities^32^. Thus, while the absolute uptake in our AI cohort is lower than that obtained with intensive navigation, the three‑fold relative difference compares favorably with results from other digital interventions and approximates the effect sizes reported in labor‑intensive, care-coordination interventions^33–35^.

Retention in PrEP care—defined as documentation of at least one follow-up visit or prescription refill—was significantly higher among individuals who interacted with the AI chatbot compared with those who did not. In a mobile health application trial among Tanzanian PrEP users, 86% completed at least one follow-up visit within three months, and 72% remained engaged at six months^34^. Likewise, a real-time digital intervention among men who have sex with men achieved 83% adherence at six months compared with 60% among controls^35^. However, it must be noted that these studies and ours differ in follow-up duration, geographic context, and methodology. Moreover, a global meta-analysis estimated that fewer than one-third of PrEP initiators maintained prevention-effective persistence by six months (i.e., discontinued or suboptimal use among ~67.0% of participants)^36^. The 57.0% follow-up rate among conversational AI users in our study is comparable to short-term retention outcomes reported in other digital PrEP interventions, though these comparisons should be interpreted cautiously given differences in study design and follow-up duration. Additionally, the mean follow-up interval was approximately two weeks shorter among AI-users, illustrating an opportunity to accelerate timely follow-up in real-world contexts. While statistically significant, further studies may be necessary to establish the impact of this on clinical outcomes. Further, it is worth noting that although the AI program substantially improved PrEP initiation and follow-up compared with standard care, overall engagement and persistence remain less than perfect. This mirrors national patterns in PrEP utilization, where structural, behavioral, and social barriers continue to limit uptake and adherence despite expanded access.

Engagement metrics indicated sustained, bidirectional use of the AI platform. To contextualize message-volume, prior digital mental-health interventions report very low two-way messaging: in one review, only 8.5% of participants sent more than five messages over 14 weeks, and most (56.4%) sent none^37^. The preference by 80% of users for the “Glitter Byte” persona underscores the potential appeal of culturally resonant and entertaining dialog styles in sustaining engagement. Although we did not assess the specific reasons for persona choice, these data suggest that approachable and identity-affirming conversational styles may help reduce stigma and encourage continued interaction^38^. While further qualitative studies are needed, engagement with the application may illustrate public interest in engaging with a generative AI model with conversational guidance rather than traditional static educational materials, which are not tailored to individual contexts.

Clinical staff additionally reported time-savings related to routine communications following AI implementation, paralleling findings that health-care AI tools can automate administrative tasks—such as appointment scheduling and medication reminders—and thereby reduce staff workload (testing staff, clinic managers, PrEP navigators, care providers)^39^. Systematic reviews of conversational agents in health care highlight efficiency gains, noting that chatbots can manage high volumes of inbound inquiries without added staff burden, effectively serving as first-line navigators for scheduling, prescription refills, and common clinical questions^24^. No adverse events or misinformation episodes attributable to the chatbot were documented, aligning with safety assessments of healthcare chatbots that report minimal unintended harms when human-in-the-loop oversight is employed^38^. These operational efficiencies may enable reallocation of personnel time toward complex clinical tasks, enhancing overall care capacity and quality.

This analysis offers several strengths. It examines one of the largest real-world deployments of a conversational AI system within HIV prevention services across a geographically and demographically diverse network. The study uses routinely collected, de-identified operational data, providing a pragmatic view of how such technology functions in everyday care delivery. By characterizing short-term engagement and follow-up behaviors, the work contributes early, practice-based evidence on the feasibility of integrating AI-enabled communication into existing PrEP service models and reaching populations historically underrepresented in digital health research.

We additionally acknowledge several limitations. First, the study design was observational; individuals were not randomized to AI exposure. It is likely that individuals who chose to engage with the AI were inherently more motivated or health-seeking than those who did not, which could confound outcomes. Additionally, because the AI system was embedded in routine digital workflows for all eligible patients, it was not possible to determine whether initiation decisions were directly influenced by AI engagement. Accordingly, this study describes associations between AI interaction and PrEP outcomes, not causal effects. We attempted to mitigate this by including all those receiving care in the AHF Wellness network. Nonetheless, unmeasured confounders (e.g., risk perception, health literacy) could influence both AI use and PrEP outcomes. Of note, because AHF Wellness Clinics provide low- or no-cost PrEP regardless of insurance status, health-insurance coverage was not consistently captured in the dataset and could not be incorporated into the current analysis. Given this analysis used aggregate, de-identified operational data without consistent individual-level covariates across sites, multivariable adjustment was not feasible and would not have produced reliable estimates of independent associations. Accordingly, a prospective analysis using linked longitudinal data across time points to evaluate temporal trends and individual trajectories by demographic subtypes may be a novel addition to the literature. Socioeconomic indicators, detailed exposure type, residency, and HIV outcome data were not available in de-identified extracts; future work linking clinic records with census or surveillance data could help address these gaps. Second, our outcome definitions, while pragmatic, were binary and did not capture nuances such as level of PrEP persistence (drug levels) or long-term PrEP continuation beyond one follow-up. Future analyses with longer follow-up will be valuable to assess whether the AI’s impact persists over multiple years and whether it affects ultimate HIV incidence in the cohort. Third, there may have been secular trends during 2024–2025 (e.g., increased overall emphasis on PrEP in the healthcare system, or COVID-19-related disruptions) that affected PrEP outcomes for all individuals, independent of the AI. However, since our comparison groups were contemporaneous, such trends would likely influence both groups similarly.

Collectively, these findings demonstrate the feasibility of implementing an AI-augmented communication platform to support engagement across multiple stages of the PrEP care continuum. Our results indicate that such tools may help improve early uptake and follow-up in real-world prevention settings, offering a scalable approach to strengthen existing HIV prevention infrastructure. These associations suggest that conversational AI could complement clinician- and community-led efforts to reduce barriers to preventive care and potentially avert new HIV infections. Future research should include randomized and longitudinal designs, cost-effectiveness analyses, and qualitative evaluations to assess sustainability, user experience, and long-term clinical outcomes.

### Ethics approval

This study was reviewed by the Advarra Institutional Review Board, which determined that the project does not meet the definition of human subjects research under 45 CFR 46 and is therefore exempt from IRB oversight (Protocol ID: Pro00084144, determination dated January 3, 2025). The study involved retrospective analysis of de-identified data and did not involve contact with participants. Informed consent was not required.

## Supplementary information

Supplementary Information

## Data Availability

No custom algorithms were developed for this study. Data processing and statistical analyses were performed using base R (version 4.4.2) and standard packages (including tidyverse, dplyr, and stats) with default parameters unless otherwise specified. The analysis scripts used for data cleaning, descriptive statistics, and outcome calculations are available from the corresponding author upon reasonable request and subject to institutional data-use agreements.

## Code availability

No custom code was used for data analysis beyond standard statistical packages in R. Code used for data cleaning and analysis is available from the corresponding author upon reasonable request. No custom algorithms were developed for this study. Data processing and statistical analyses were performed using base R (version 4.4.2) and standard packages (including *tidyverse*, *dplyr*, and *stats*) with default parameters unless otherwise specified. The analysis scripts used for data cleaning, descriptive statistics, and outcome calculations are available from the corresponding author upon reasonable request and subject to institutional data-use agreements.
