# Supplementary material for: AI-augmented communication improves HIV PrEP initiation and persistence in populations disproportionately impacted by HIV
Source: NPJ Digit Med. 2026 Mar 7;9:411. doi: 10.1038/s41746-026-02519-3 (PMC13223211; doi:10.1038/s41746-026-02519-3)
Supplement: Supplementary file 1 — Supplementary Information [file 41746_2026_2519_MOESM1_ESM.pdf]

Supplementary Information

Accuracy	Ratings	Percent
1	9	0.01
2	843	0.87
3	3,080	3.17
4	4,486	4.62
5	88,662	91.33
**Total**	**97,080**	**100%**

Comprehensiveness	Ratings	Percent
1	8	0.01
2	521	0.54
3	4,720	4.86
4	4,443	4.58
5	87,386	90.02
**Total**	**97,078**	**100%**

Contextual Sensitivity	Ratings	Percent
1	7	0.01
2	865	0.89
3	4,715	4.86
4	3,945	4.06
5	87,548	90.18
**Total**	**97,080**	**100%**

Supplemental Table 1: Clinician Ratings of AI-Generated Chatbot Responses Across Three Quality Domains. Healthvana staff, trained on a standardized assessment rubric and certified through the University of Washington's National STD and HIV PrEP Curricula, evaluated 97,080 AI-generated messages using a 5-point scale assessing accuracy, comprehensiveness, and contextual sensitivity. Across all domains, the majority of responses received a rating of 5: 91.3% of responses were rated 5 for accuracy, 90.0% for comprehensiveness, and 90.2% for contextual sensitivity. Fewer than 1% of responses received scores of 1 or 2.
